# SMALL INTESTINE GASTROINTESTINAL STROMAL TUMOR MASQUERADING AS UTERINE MALIGNANCY

**DOI:** 10.51894/001c.122864

**Published:** 2024-08-30

**Authors:** Brandon Wiggins, Mark Rigby, Elaine Ognjanovsk, Mark Minaudo

**Affiliations:** 1 Gastroenterology Ascension Genesys

## 23

### INTRODUCTION

Gastrointestinal stromal tumors (GISTs) constitute <1% of gastrointestinal tumors, with extra-gastrointestinal stromal tumors (EGIST) comprising <10% of all GISTs. We present the rare case of a 68-year-old female, with a preoperatively mistaken uterine carcinoma, diagnosed as a primary EGIST located in the posterior cul-de-sac connected to the jejunum.

### CASE DESCRIPTION

A 68-year-old female underwent an elective Roux-en-Y gastric bypass. Over the following months, postoperative complications included peritonitis, anastomotic leak, septic shock, liver abscesses, intractable nausea and vomiting. Incidentally, computed tomographic imaging identified a mass in the uterus. Magnetic resonance imaging demonstrated a right hemipelvis mass thought to originate from the right adnexa. Cervical biopsy, pap smear and tumor panels were unremarkable. The patient was scheduled for total abdominal hysterectomy (TAH) and intraoperatively, a 15 cm mass was found to be connected to the jejunum, in the posterior cul-de-sac. Small bowel resection with biopsies was performed in lieu of the planned TAH. Biopsies stained positive for CD117, DOG-I, and SMA consistent with GIST. The patient was then started on imatinib and eventually discharged in stable condition.

### DISCUSSION

Although rare, GISTs are the most common mesenchymal neoplasm of the GI tract and have a near-universal expression of the CD117 antigen. They originate from the interstitial cells of Cajal and typically present as a subepithelial, intraluminal mass. Tumors are most commonly identified in the stomach (40-60%) followed by the jejunum and ileum (25-30%). EGISTs are rare, and if found, are usually associated with metastases from an undetected intra-gastrointestinal tumor. According to a recent report, <30 cases of GISTs were preoperatively mistaken for gynecological neoplasms. Our case is a unique addition to the existing literature. A combination of surgical resection and adjuvant chemotherapy is recommended in treatment of GISTs. Imatinib remains one of the mainstay therapies for most GISTs. EGISTs are a rare and aggressive group of stromal tumors and should be included in the differential diagnosis of a patient presenting with an extra-luminal abdominal mass. Imaging fails to identify presumed gynecological neoplasms as GISTs. It is prudent to consider non-gynecological tumors for pelvic masses.

